# Integrative pan-cancer landscape of MMS22L and its potential role in hepatocellular carcinoma

**DOI:** 10.3389/fgene.2022.1025970

**Published:** 2022-10-06

**Authors:** Zhiting Guo, Fahui Liu, Qiming Gong

**Affiliations:** ^1^ College of Biological Science and Engineering, Fuzhou University, Fuzhou, China; ^2^ Department of Medical Biochemistry and Cell Biology, Institute of Biomedicine, University of Gothenburg, Gothenburg, Sweden; ^3^ Department of Nephrology, Affiliated Hospital of Youjiang Medical University for Nationalities, Baise, Guangxi, China

**Keywords:** MMS22L, pan-cancer, prognosis, immunotherapy, hepatocellular carcinoma

## Abstract

Methyl methanesulfonate-sensitivity protein 22-like (MMS22L) is crucial in protecting genome integrity during DNA replication by preventing DNA damage and maintaining efficient homologous recombination. However, the role of MMS22L in human cancers remains unclear. Here, we reported the landscape of MMS22L using multi-omics data and identified the relationship between the MMS22L status and pan-cancer prognosis. In addition, the correlation of MMS22L mRNA expression levels with tumor mutational burden, microsatellite instability, homologous recombination deficiency, and loss of heterozygosity in pan-cancer was also described in this study. Furthermore, this study was the first to characterize the relationship between mRNA expression of MMS22L and immune cell infiltration in the tumor microenvironment in human cancer. Concurrently, this study explored the crucial role of MMS22L in different immunotherapy cohorts through current immunotherapy experiments. Eventually, we investigated the role of MMS22L in hepatocellular carcinoma (HCC). The results demonstrated that MMS22L is widely expressed in multiple HCC cell lines, and our results emphasized that MMS22L was involved in HCC progression and affects the prognosis of patients with HCC through multiple independent validation cohorts. Collectively, our findings reveal the essential role of MMS22L as a tumor-regulating gene in human cancers while further emphasizing its feasibility as a novel molecular marker in HCC. These findings provide an essential reference for the study of MMS22L in tumors.

## Introduction

Methyl methanesulfonate-sensitivity protein 22-like (MMS22L) is widely known for its essential role in DNA replication, which involves the formation of the MMS22L-TONSL protein complex (Duro et al., 2010; Saredi et al., 2016). This complex protects DNA molecules in the replication phase and assists in the necessary DNA damage repair by participating in homologous recombination (HR). Since tumor cells divide and differentiate significantly more frequently than normal cells, they have a longer “sensitive period” and a great demand for DNA damage repair (Miller et al., 2019). Therefore, cancer cell DNA is more fragile than normal cell DNA, especially during division, thereby requiring more protection. This finding suggests that MMS22L may serve as a new drug target for blocking DNA repair in cancer cells. The interaction between MMS22L and NFKBIL2 has been proposed as another mechanism for participating in the NFKB pathway in cancer cells (Nguyen et al., 2012), further identifying it as a promising target for cancer therapy. Therefore, these findings suggest that the activity of MMS22L or its complexes involved in tumor cell proliferation appears to be essential in tumorigenesis.

Although MMS22L is an attractive target for cancer therapy, its application in tumor therapy still faces numerous problems. Some studies have demonstrated the complex role of MMS22L in different types of tumors. MMS22L, for instance, was detected in lung cancers as an oncogene involved in tumor proliferation and metastasis (Nguyen et al., 2012). Conversely, a lower expression of MMS22L was associated with worse clinical outcomes in esophageal squamous cell carcinoma, suggesting MMS22L is a tumor suppressor (Luo et al., 2021). Concurrently, many reports described that MMS22L affected the prognosis of patients by regulating drug sensitivity during treatment (Liu et al., 2021). Altogether, these results suggest that the roles of MMS22L in different tumors are diverse and complex and suggest that MMS22L may be a promising target for tumor therapy. However, tumor heterogeneity limits our knowledge about the role of MMS22L in human tumors. Therefore, a comprehensive understanding of the molecular characterization and clinical relevance of MMS22L in human cancer is urgent. Understanding the aberrant expression and genomic alterations of MMS22L may help to clarify its role in cancer prognosis and treatment.

This study presents a relatively comprehensive assessment of MMS22L in pan-cancer using public repository data. The results of this study demonstrate the status of MMS22L and its association with prognostic data and genomic heterogeneity of tumor patients. In addition, we are the first to analyze the association of MMS22L with the immune system in pan-cancer and its role in immunotherapy. Further, by combining multi-cohorts and molecular biology experiments, this study identifies the vital role and clinical significance of MMS22L in hepatocellular carcinoma (HCC). Overall, this study is the first to reveal the landscape of MMS22L in pan-cancer and preliminarily explores the potential role of MMS22L in HCC. These findings demonstrate the potential value of MMS22L in cancer and lay the foundation for developing MMS22L in clinical applications.

## Materials and methods

### Data source

The UCSC Xena database (https://xenabrowser.net/datapages/) was used to download the gene expression data and corresponding clinical information of The Cancer Genome Atlas (TCGA) and Genotype-Tissue Expression (GTEx). Copy number variation (CNV) data was processed through GISTIC2.0, and single-nucleotide variants (SNV) data were also downloaded. cBioPortal (http://cbioportal.org) was used to visualize the frequency of genomic alterations of MMS22L in 33 cancer types. The Human Protein Atlas (https://www.proteinatlas.org/) database was used to confirm MMS22L protein localization at the cellular level. MMS22L protein interaction information was obtained from ComPPI database (http://comppi.linkgroup.hu). Abbreviations for cancers mentioned in this study are shown in Supplementary Table S1. The expression level of MMS22L in different immunotherapy cohorts, the relationship between MMS22L expression and prognosis of patients with immunotherapy, and MMS22L expression and drug sensitivity were analyzed using the Biomarker Exploration of Solid Tumors (BEST) web server (https://rookieutopia.com/). To further explore the effect of MMS22L in HCC, four microarray data of patients with HCC, including GSE25097, GSE22058, GSE36376, and GSE54236, were downloaded from the Gene Expression Omnibus (GEO). RNA sequencing (RNA-seq) data of ICGC-LIRI-JP were downloaded from the International Cancer Genome Consortium (ICGC) portal (https://dcc.icgc.org/projects/LIRI-JP). In addition, RNA-Seq data of the LIHC-CN cohort was obtained from a previous study (Gao et al., 2019).

### Cell culture and RT-qPCR

Huh-7, 7721, MHCC97H, SK, and LO2 cells were cultured in Dulbecco’s Modified Eagle Medium (DMEM) media supplemented with 10% fetal bovine serum (FBS, Gibco) at 37 °C with 5% CO_2_. SNU398 and SNU449 cells were cultured in 1640 media supplemented with 10% FBS (Gibco) at 37°C with 5% CO_2_. Hep3B and HepG2 cells were cultured in Minimum Essential Medium (MEM) media supplemented with 10% FBS (Gibco) at 37 °C with 5% CO_2_. For RT-qPCR experiments, Trizol reagent (Thermo Fisher Scientific) and RNeasy Lipid Tissue Kit (QIAGEN) were used to isolate total RNA according to the manufacturer’s instructions, and cDNA was synthesized using reverse transcriptase (Promega). The sequences of primers used in this study are listed in Supplementary Table S2. Sangon Biotech, China, synthesized all primers.

### Protein extraction and western blotting

All cells were lysed in RIPA buffer (Sigma-Aldrich) containing a 1% protease inhibitor cocktail (Thermo Fisher Scientific) and phosphatase inhibitor cocktail (Thermo Fisher Scientific). The MMS22L protein and anti-beta actin were separated using 10% SDS–PAGE and transferred to polyvinylidene difluoride membranes. After blocking the membranes with 5% BSA in 1 × PBST buffer (10 mM phosphate buffer, 2.7 mM KCl, 137 mM NaCl, 0.05% Tween-20; pH 7.4) at room temperature for 1h, the membranes were separately incubated overnight with MMS22L recombinant monoclonal antibody (1:1,500; ab181047, Abcam) and anti-beta actin (1:1,500; ab8227, Abcam) at 4°C. The membranes were washed with 1 × PBST buffer three times to remove the unbound protein.

### Survival analysis of MMS22L in pan-cancer

A sample barcode was used to merge CNV and clinical survival data for MMS22L in pan-cancer. Tumor samples were divided into wild-type (WT), amplification (Amp.), and deletion (Dele). groups. Log-rank tests were performed to evaluate the survival difference between groups. The SNV data and clinical survival data were merged by sample barcode for survival analysis of MMS22L in pan-cancer studies. Tumor samples were divided into mutant groups when the specific genes in these samples were mutated (deleterious mutants). Cox proportional hazards models and log-rank tests were used to evaluate the survival difference between WT and mutant groups. For survival analysis of MMS22L mRNA expression in pan-cancer, mRNA expression and survival data were merged by sample barcode, and the median value of MMS22L was used to divide tumor samples into high and low expression groups. Next, we used the R package survival to fit the survival time and survival status within the two groups. Cox proportional hazards models and log-rank tests were used to analyze the correlation between mRNA expression of MMS22L with overall survival (OS), disease-specific survival (DSS), disease-free interval (DFI), and progression-free interval (PFI) in pan-cancer.

### Analysis of genomic heterogeneity

Homologous recombination deficiency (HRD) data (Thorsson et al., 2018), microsatellite instability (MSI) scores (Bonneville et al., 2017), and loss of heterozygosity (LOH) data (Thorsson et al., 2018) were collected from previous studies. SNV data processed by MuTect2 were from Genomic Data Commons (GDC) (https://portal.gdc.cancer.gov/). With the R package “maftools”, the tumor mutational burden (TMB) value was calculated for each tumor.

### Gene set enrichment analysis

To determine the pathways associated with MMS22L, for each tumor type, samples were divided into the top 30% and bottom 30% groups based on the expression level of MMS22L. The hallmark gene set (h.all.v7.2. symbols) was downloaded from MSigDB (https://www.gsea-msigdb.org/gsea/msigdb/). Then, the gene set enrichment analysis (GSEA) was performed using R software.

### Correlation analysis of MMS22L and immune cell infiltration

The correlation between MMS22L mRNA expression and immune cell infiltration in pan-cancer was downloaded from the TIMER2 database (http://timer.cistrome.org/). Nineteen immune cells, including B cells, cancer-associated fibroblasts, CD4^+^ T cells, CD8^+^ T cells, myeloid dendritic cells, endothelial cells, eosinophils, gamma delta T cells, hematopoietic stem cells, macrophages, mast cells, myeloid-derived suppressor cells, monocytes, neutrophils, natural killer (NK) cells, NK T cells, common myeloid progenitors, regulatory T cells, and follicular helper T cells, were incorporated in the present study.

### Statistical analysis

In addition to the aforementioned bioinformatic tools, GraphPad Prism 9.0 and R software (4.2.1, www.r-project.org) were used to perform analyses in this study. Comparisons between continuous variables were made using Wilcoxon rank-sum test. The association between two continuous variables was assessed using Spearman’s rank correlation. *p* < 0.05 was considered statistically significant in this study.

## Result

### Basic information about MMS22L in pan-cancer

**FIGURE 1:**
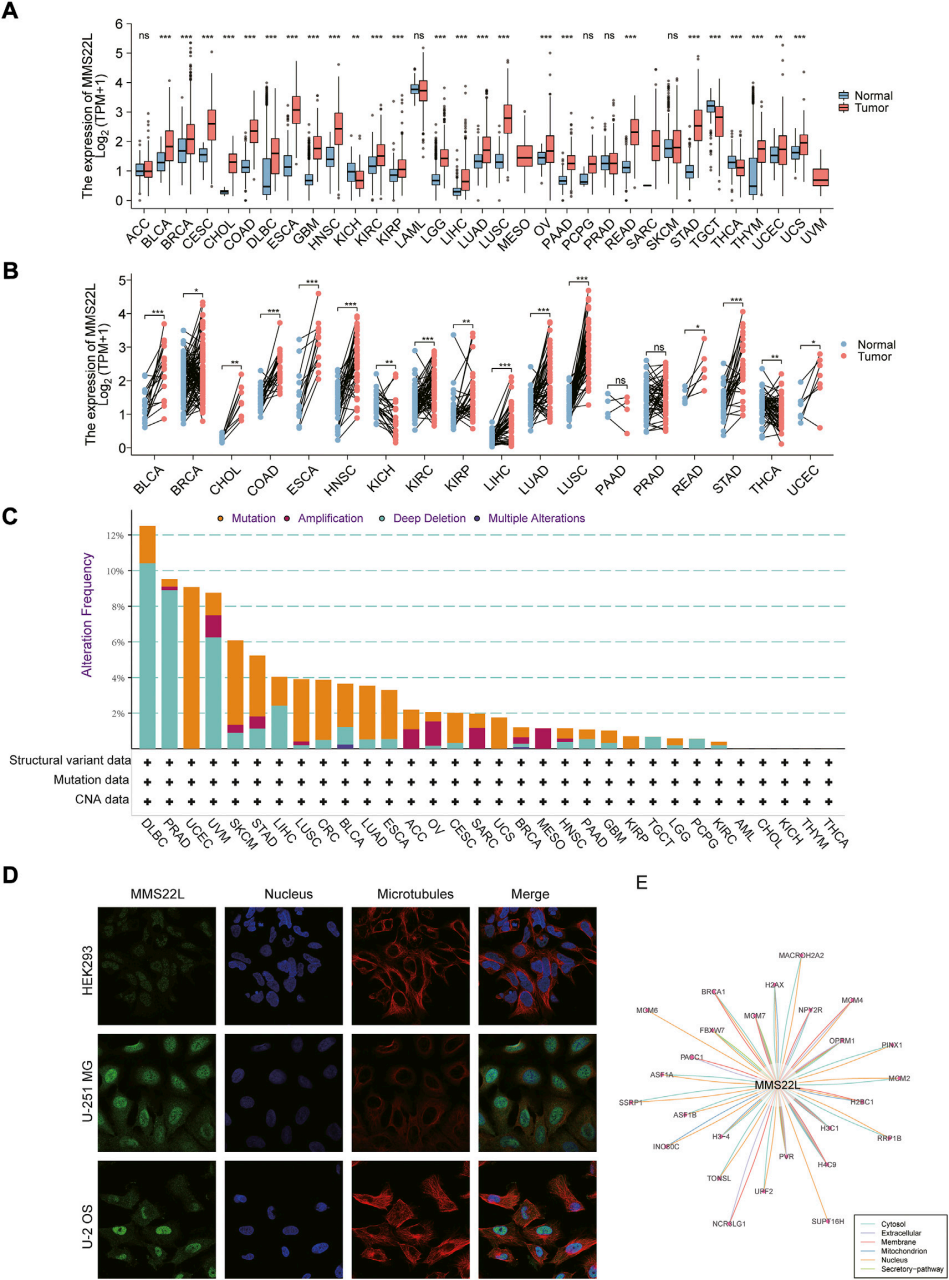
Basic information about methyl methanesulfonate-sensitivity protein 22-like (MMS22L) in pan-cancer. **(A)** Differential expression of MMS22L based on The Cancer Genome Atlas and Genotype-Tissue Expression databases in pan-cancer. **(B)** Paired analysis of MMS22L expression in pan-cancer. **(C)** The landscape of MMS22L genomic alterations in pan-cancer according to the cBioPortal database. **(D)** Immunofluorescence images depicting localization information of MMS22L protein in HEK 293, U-2 OS, and U251 MG cell lines. **(E)** Protein-protein interaction network formed by MMS22L. ns, *p* ≥ 0.05; *, *p* < 0.05; **, *p* < 0.01; ***, *p* < 0.001.

To gain a basic understanding of MMS22L in pan-cancer, we used the TCGA and GTEx databases to evaluate the expression level of MMS22L in cancer compared with that in normal tissues. High expression levels of MMS22L were detected in most cancer types, including BLCA, BRCA, CESC, CHOL, COAD, DLBC, ESCA, GBM, HNSC, KIRC, KIRP, LGG, LIHC, LUAD, LUSC, OV, PAAD, READ, STAD, THYM, UCEC, UCS. The expression levels of MMS22L in TGCT, THCA, and KICH were lower than in normal tissues (Figure 1A). The results of the paired analysis showed that MMS22L still had significant differences among BLCA, BRCA, CHOL, COAD, ESCA, HNSC, KIRC, KIRP, LIHC, LUAD, LUSC, READ, STAD, UCEC, TGCT, and KICH (Figure 1B). Analysis of genomic alterations in MMS22L showed that there was no universal alteration of MMS22L in pan-cancer. DLBC was the most commonly altered cancer type, and among the mutant types was primarily deep deletion, which occurred in approximately 10% of DLBC patients. It is noteworthy that somatic mutation was the only genomic event in UCEC (Figure 1C). Images captured with immunofluorescence showed that MMS22L protein was predominantly localized and distributed in the nucleus of HEK293, U-251 MG, and U-2 OS tumor cell lines (Figure 1D). Analysis of protein-protein interaction networks revealed that proteins closely related to MMS22L and their subcellular localization were distributed in the cytosol, mitochondrion, extracellular membrane, nucleus, and secretory pathway (Figure 1E).

### Correlation of MMS22L with survival in different omics pan-cancer data

**FIGURE 2:**
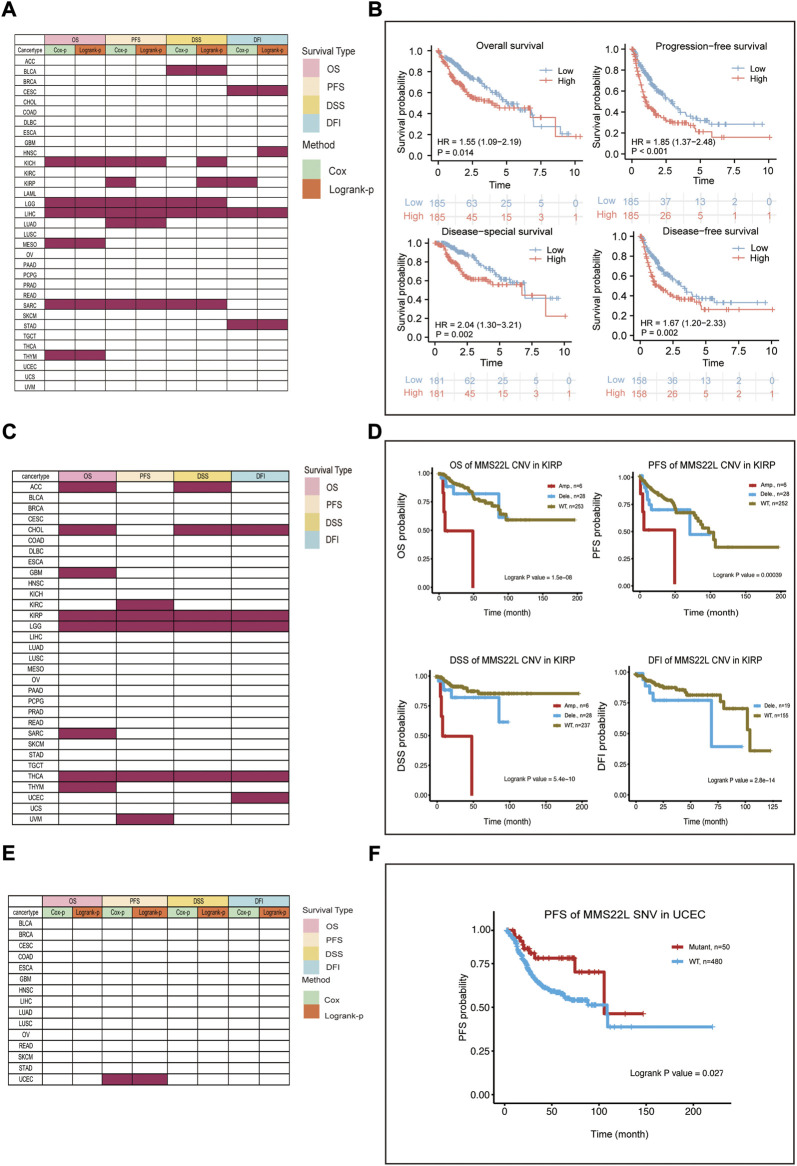
Survival landscape of methyl methanesulfonate-sensitivity protein 22-like (MMS22L) in pan-cancer. **(A)** Summary of the correlation between mRNA expression of MMS22L with OS, DSS, DFI, and PFI based on the univariate Cox regression and log-rank tests in pan-cancer. **(B)** High mRNA expression of MMS22L was associated with worse outcomes in patients with HCC. **(C)** Summary of the correlation between the copy number variation status of MMS22L using OS, DSS, DFI, and PFI based on the log-rank tests. **(D)** Amplification of MMS22L is associated with a worse prognosis in kidney renal papillary cell carcinoma. **(E)** Summary of the correlation between the single-nucleotide variant status of MMS22L and OS, DSS, PFI, and DFI based on the univariate Cox regression and log-rank tests. **(F)** Patients with mutated MMS22L had a better prognosis of progression-free survival in uterine corpus endometrial carcinoma.

We further analyzed the association of data from different omics with four different clinical prognostic outcomes in pan-cancer. The specific analysis is indicated in Supplementary Table S3. Specifically, the expression level of MMS22L was significantly correlated with the prognosis of KICH, LGG, LIHC, MESO, SARC, and THYM (Figure 2A). Among them, we found that only in LIHC was the expression level of MMS22L significantly associated with the four clinical outcomes using two different methods. We further visualized the correlation of MMS22L expression with different clinical outcomes, and the results showed that a higher expression of MMS22L was associated with a worse prognosis in patients with HCC (Figure 2B). The CNV level of MMS22L was associated with overall survival (OS) in adrenocortical carcinoma (ACC), CHOL, GBM, KIRP, LGG, SARC, THYM, and THCA (Figure 2C). Meanwhile, the expression of MMS22L was correlated with KIRC, KIRP, LGG, THCA, and UVM. It was evident that the expression of MMS22L in KIRP, LGG, and THCA was associated with all four outcomes. We further visualized the relationship between MMS22L CNV in KIRP and the four types of clinical outcomes. The analysis showed that patients with wild-type MMS22L showed better survival in all clinical outcomes in KIRP (Figure 2D). Finally, we analyzed the association of the SNV status of MMS22L with patient prognosis. We observed that only mutations of MMS22L were associated with the survival of patients with UCEC (Figure 2E). Furthermore, mutations of MMS22L were tightly associated with better progression-free survival of patients with UCEC (Figure 2F).

### Functional analysis of MMS22L in pan-cancer

**FIGURE 3:**
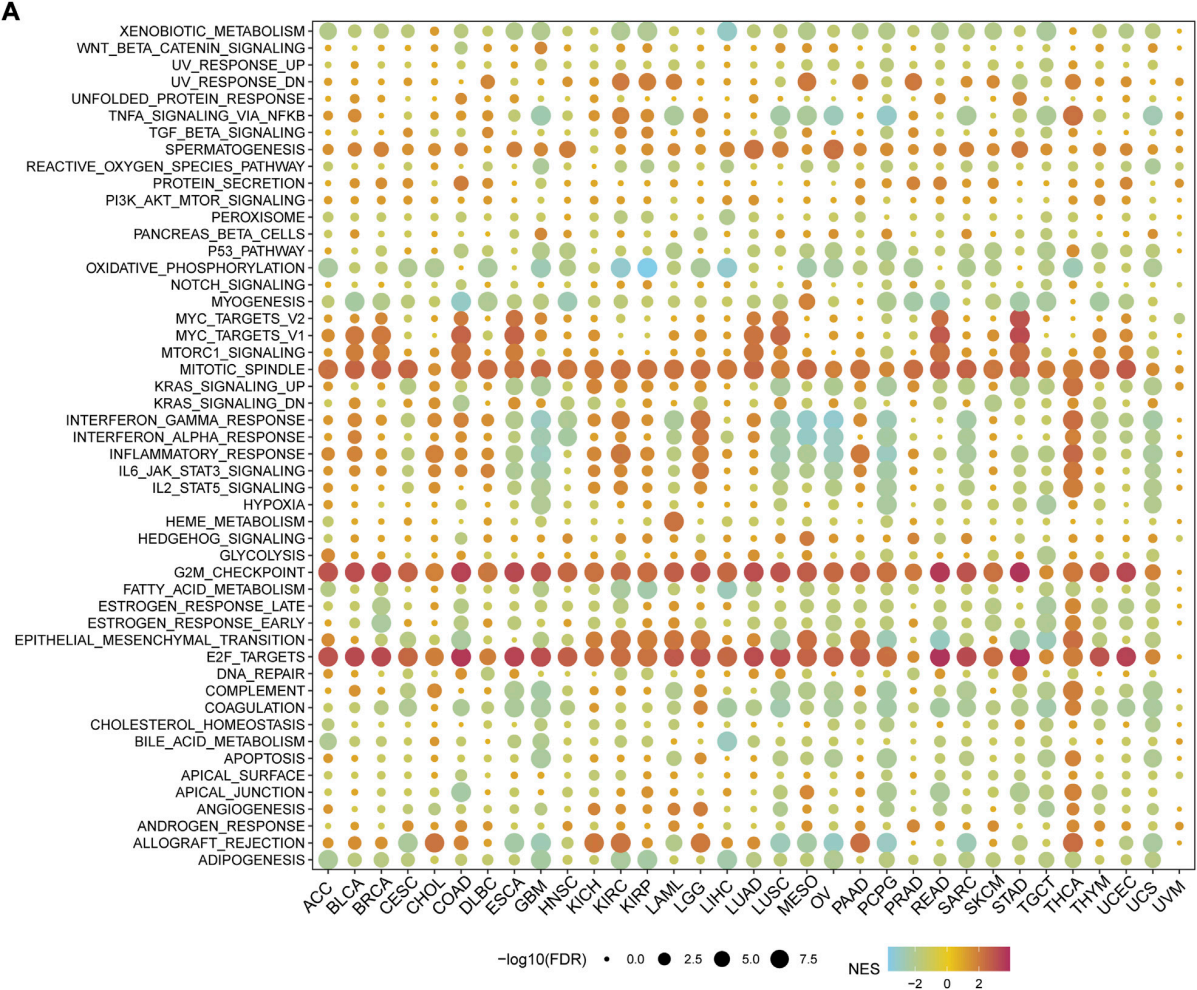
Functional analysis of methyl methanesulfonate-sensitivity protein 22-like (MMS22L) in pan-cancer. **(A)** Enrichment analysis for hallmark gene sets between high- and low- MMS22L expression groups.

To elucidate the potential mechanism of action of MMS22L, we further analyzed the pathways involved in MMS22L using the GSEA function in pan-cancer (Figure 3A). The analysis showed that pathways including mitotic spindle, G2M checkpoint, and E2F targets were significantly activated in the MMS22L high expression group in pan-cancer. The above results suggested that MMS22L played a key role in the cell cycle in human tumors, which might be one of the key factors for MMS22L to affect tumor evolution. In addition, we noticed that immune pathways, such as TNFα-signaling through NF-kB, inflammatory response, IFN-α response, and IFN-γ response were significantly activated in GBM in the MMS22L low expression group. Conversely, these pathways were significantly activated in the MMS22L high expression group in THCA. These results suggest that the role of MMS22L in pathways involved in immune responses differed across tumor types.

### Correlation analysis of MMS22L with genomic heterogeneity in pan-cancer

**FIGURE 4:**
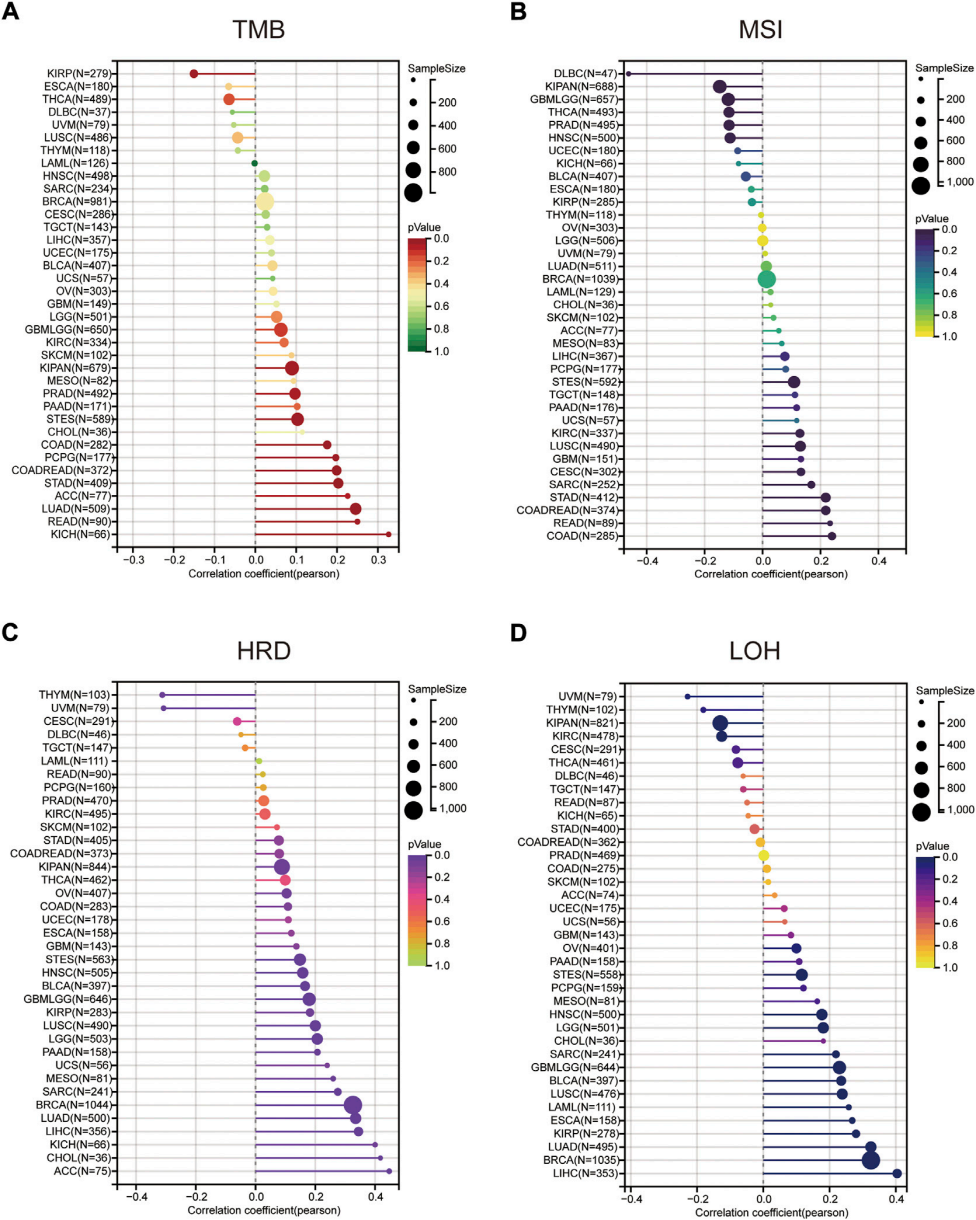
The correlation between methyl methanesulfonate-sensitivity protein 22-like (MMS22L) expression and genomic heterogeneity in pan-cancer. **(A)** Spearman correlation analysis of tumor mutational burden and MMS22L mRNA expression. **(B)** Spearman correlation analysis of microsatellite instability and MMS22L mRNA expression. **(C)** Spearman correlation analysis of homologous recombination deficiency and MMS22L mRNA expression. **(D)** Spearman correlation analysis of loss of heterozygosity and MMS22L mRNA expression.

We further explored the relationship between MMS22L expression and genomic heterogeneity. Genomic heterogeneity has been increasingly reported to be related to numerous genes, although unclear. The relationship between various types of genomic heterogeneity, including TMB, MSI, HRD, and LOH and MMS22L expression in pan-cancer are still unclear. After integrating TMB data and MMS22L expression in pan-cancer, the TMB of 11 types of tumors (LUAD, COAD, READ, STES, KIPAN, STAD, prostate adenocarcinoma (PRAD), pheochromocytoma and paraganglioma (PCPG), ACC, and KICH) were positively related to MMS22L expression. However, the TMB of KIRP was negatively correlated with MMS22L expression (Figure 4A). The MSI scores were closely related to 15 types of tumors. CESC, COAD, COADREAD, STES, SARC, STAD, KIRC, LUSC, and READ were positively related to MMS22L expression (Figure 4B). In addition to TMB and MSI, the relationship between HRD data and the expression of MMS22L is also presented in Figure 4C. A significant correlation was observed in 20 tumors, with a significant positive correlation in 18 tumors (GBMLGG, LGG, LUAD, BRCA, STES, SARC, KIRP, KIPAN, HNSC, LUSC, LIHC, MESO, PAAD, OV, BLCA, ACC, KICH, and CHOL), and a negative correlation in two tumors (THYM and UVM). Finally, the correlation between LOH data and MMS22L expression was analyzed. In contrast, 12 types of cancers (GBMLGG, LGG, LUAD, BRCA, STES, SARC, KIRP, HNSC, LUSC, LIHC, OV, and BLCA) in which MMS22L expression was positively related to HRD had a significant positive correlation. However, there was a significant negative correlation among the three tumors, such as KIPAN, KIRC, and UVM (Figure 4D).

### MMS22L and immune cells in pan-cancer

**FIGURE 5:**
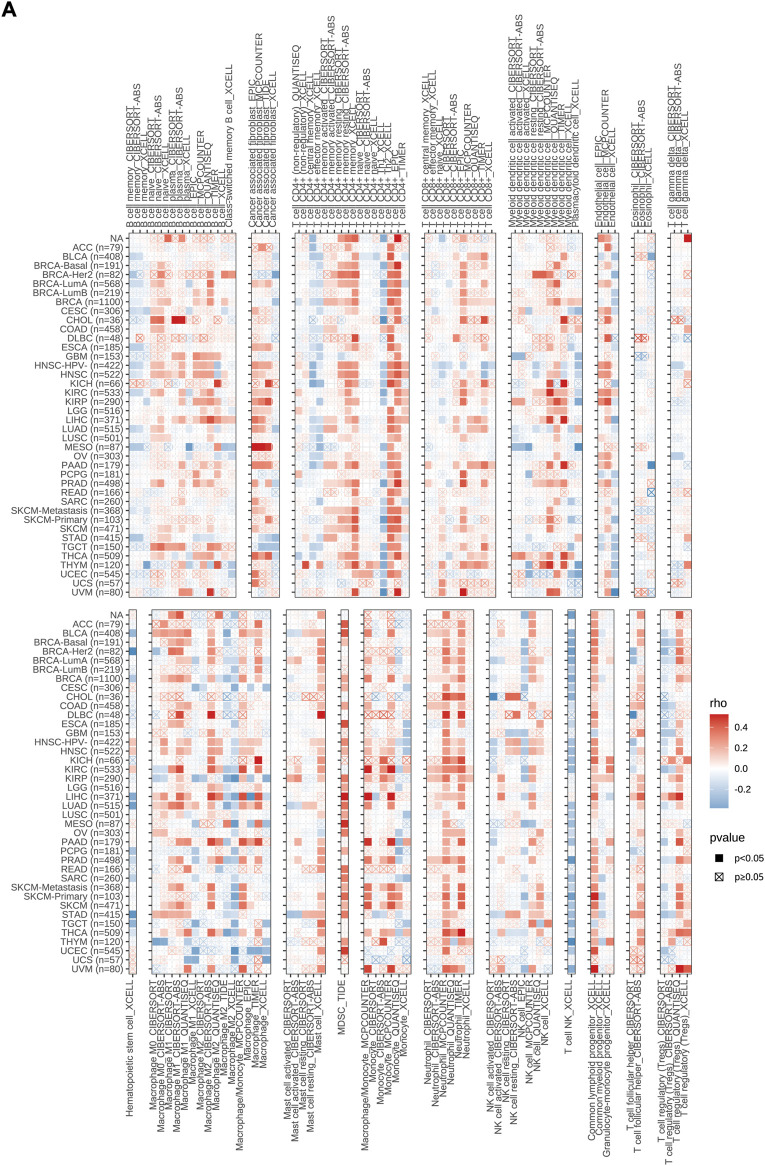
Associations of methyl methanesulfonate-sensitivity protein 22-like (MMS22L) expression with immune infiltration in pan-cancer. **(A)** The correlations of MMS22L mRNA expression and immune cell infiltration across human cancers according to the TIMER2.0 database.

In order to confirm the relationship between MMS22L expression and cancer immunity, we examined the correlation between MMS22L expression and the infiltration of immune cells in pan-cancer using the TIMER2 database (Figure 5A). The results indicated that MMS22L was positively associated with the infiltration levels of B cells, cancer-associated fibroblasts (CAF), CD4^+^ T cells, CD8^+^ T cells, dendritic cells, endothelial cells (Endo), macrophages, mast cells, myeloid-derived suppressor cells (MDSC), monocytes, neutrophils, NK cells, common lymphoid progenitor cells (CLP), T follicular helper cells (Tfh), and Tregs in the majority of these common cancers. In contrast, MMS22L was negatively associated with the infiltration levels of eosinophils (Eos), HSC, and NKT. In addition, it was also significant that almost all types of cancers were positively associated with MDSC and neutrophils. Our results indicated that MMS22L is associated with diverse cancers and may affect the effectiveness of therapy by associating with immune cells.

### Immunotherapy analyses of MMS22L

**FIGURE 6:**
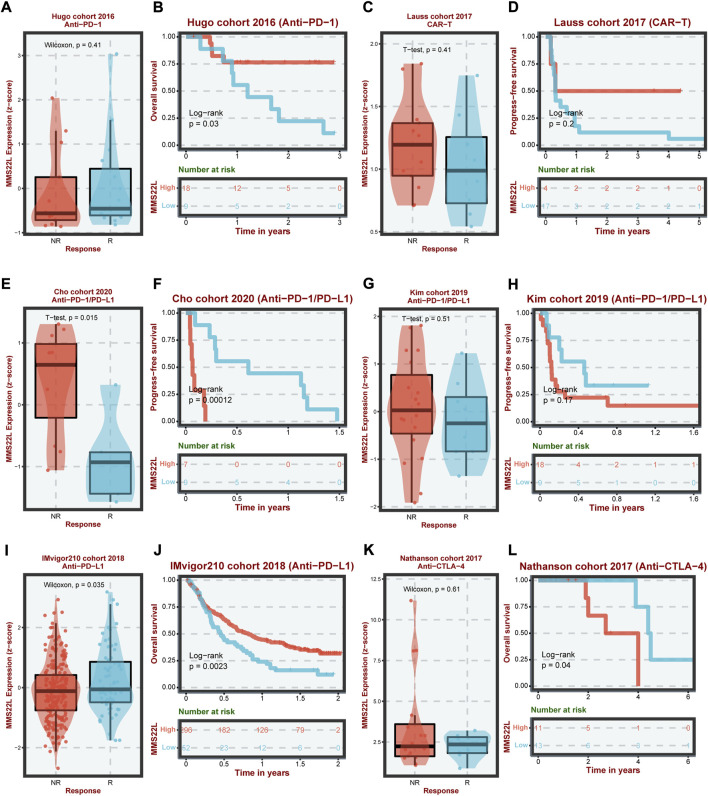
Analysis of methyl methanesulfonate-sensitivity protein 22-like (MMS22L) in cancer immunotherapy in different cohorts. The difference in MMS22L expression between the corresponding group and the non-corresponding group and its relationship with the prognosis of patients in Hugo **(A,B)**, Lauss **(C,D)**, Cho **(E,F)**, Kim **(G,H)**, IMvigor210 **(I,J)**, and Nathanson **(K,L)** cohorts.

The previous analysis showed that MMS22L had different immunoregulatory functions in different tumor types and correlated with immune cells in the tumor microenvironment. Therefore, we analyzed the expression of MMS22L in six different immunotherapy cohorts and its correlation with patient prognosis (Figures 6A–L). The analytical results showed that in the Cho cohort 2020, the expression level of MMS22L in the nonresponse (NR) group was significantly higher than that in the response (R) group, and the higher expression of MMS22L was significantly associated with a worse prognosis of patients (Figures 6E,F). Conversely, in the IMvigor210 cohort, the expression level of MMS22L in the R group was significantly higher than that in the NR group. Patients with higher levels of MMS22L showed a better clinical outcome (Figures 6I,J). However, MMS22L expression levels did not differ significantly in other immunotherapy cohorts’ responses and prognostic outcomes. These results demonstrated the feasibility of MMS22L as an immunotherapy marker. These results further suggested that our focus on heterogeneity among different tumor types might help us to better understand the differences in the effects of immunotherapy among different tumor types.

### Clinical relevance of MMS22L in HCC

**FIGURE 7:**
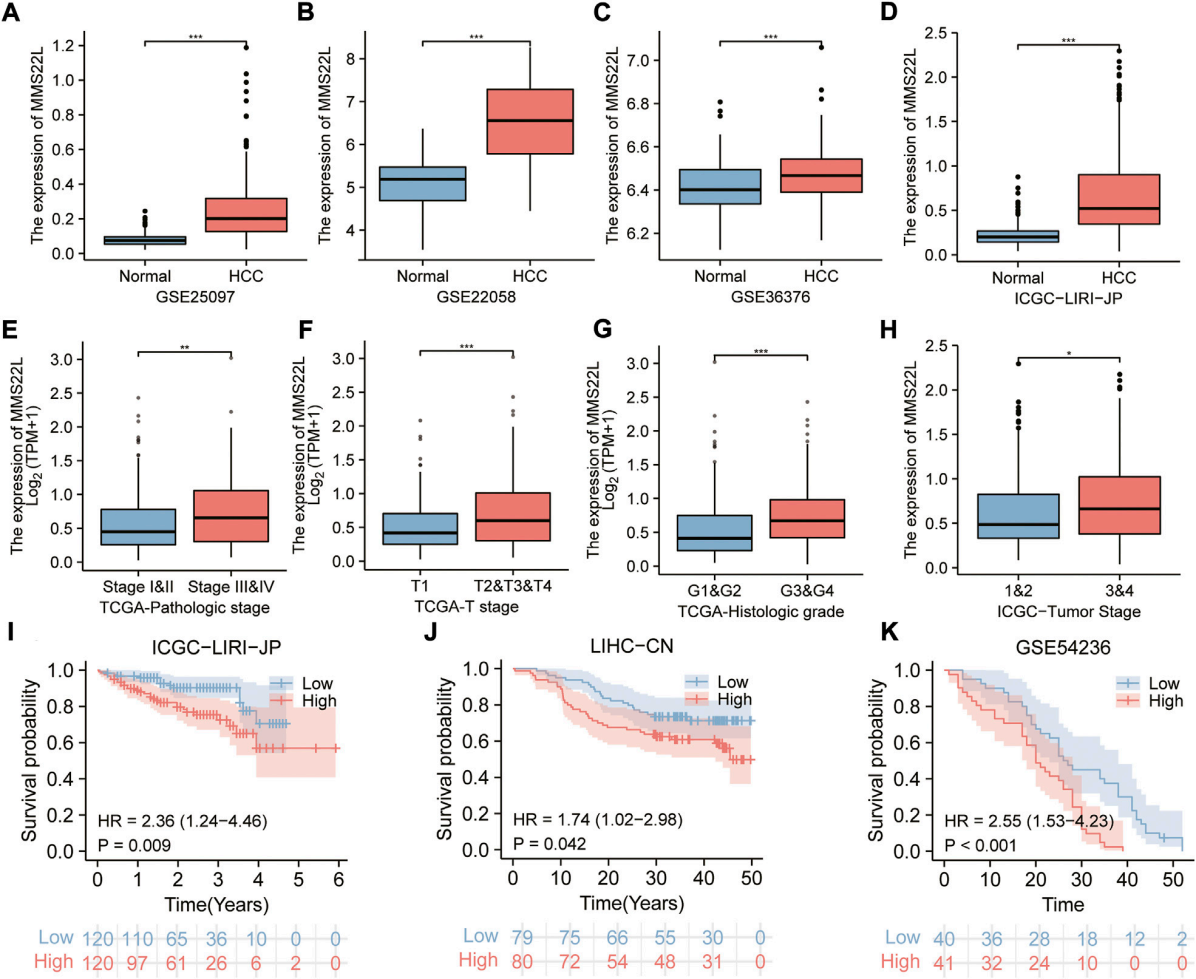
The correlation of methyl methanesulfonate-sensitivity protein 22-like (MMS22L) expression with clinical parameters in hepatocellular carcinoma (HCC). **(A–D)** Boxplot for multiple HCC cohorts showing the higher mRNA expression of MMS22L in tumor tissues than in non-tumor tissues. **(E–H)** The elevated level of MMS22L expression is associated with a higher degree of malignancy in HCC. **(I–K)** The Kaplan–Meier curves for MMS22L in the ICGC-LIRI-JP **(I)**, LIHC-CN **(J)**, and GSE54236 **(K)** cohorts. *, *p* < 0.05; **, *p* < 0.01; ***, *p* < 0.001.

The previous analysis demonstrated that MMS22L was an important risk factor for HCC. Therefore, we identified HCC as a representative cancer type for subsequent analysis. Using different clinical cohorts, we further explored the relationship between MMS22L expression levels and HCC. The expression level of MMS22L in tumor tissues was significantly higher than that in adjacent normal tissues in different HCC cohorts (Figures 7A–D). In addition, the expression level of MMS22L was higher in patients with higher HCC grades, suggesting that MMS22L participated in the clinical process and related to the malignancy of patients with HCC (Figures 7E–H). Kaplan-Meier curves for OS also showed that patients with higher expression of MMS22L had a worse OS than those with low expression of MMS22L in three different cohorts (Figures 7I,J). These results suggest an important role of MMS22L in HCC. We also detected the mRNA expression levels of MMS22L in HCC cell lines (7721, Hep3B, MHCC97H, SNU398, HepG2, Huh-7, SK, and SNU449 cells) and normal hepatocytes (LO2). The results showed that MMS22L expression in SNU398 was the highest among all these HCC cell lines, while it was lowest in HepG2 (Supplementary Figure S1). However, the expression of MMS22L was higher in all eight HCC cell lines than in LO2. Next, we analyzed the expression level of MMS22L in the protein of 9 cell lines and found that MMS22L expression was the highest in the LO2 cell and the lowest in the HepG2 cell (Supplementary Figure S2). Except for LO2 cell, MMS22L expression was similar in mRNA level and protein level. These results show that MMS22L expression differed in mRNA and protein levels in HCC cell lines.

### MMS22L for prediction of OS

**FIGURE 8:**
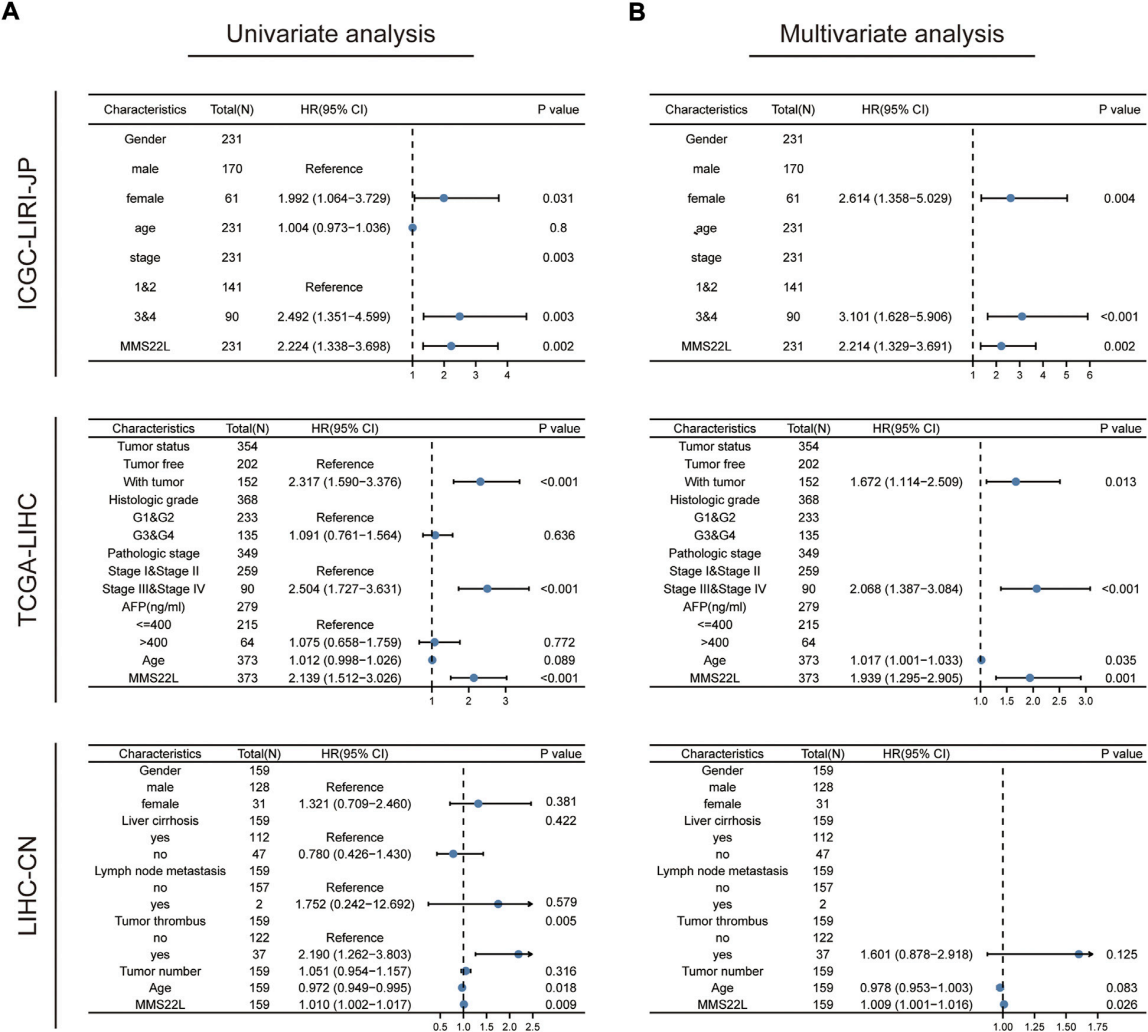
Validation of the independency of methyl methanesulfonate-sensitivity protein 22-like (MMS22L) for prediction of overall survival in patients with HCC. **(A,B)** Univariate and multivariate cox regression analysis validated that MMS22L was an independent predictor for OS in TCGA-LIHC, ICGC-LIRI-JP, and LIHC-CN cohorts.

We explored the role of MMS22L as a predictor to validate the independency of OS in patients with HCC. Univariate and multivariate cox regression analyses were used to evaluate whether MMS22L was an independent predictor of OS in TCGA-LIHC, ICGC-LIRI-JP, and LIHC-CN cohorts. As shown in Figures 8A,B, MMS22L was an independent predictor of OS in these three cohorts, which means that MMS22L played an important role in the survival of patients with HCC.

### MMS22L expression and drug sensitivity

**FIGURE 9:**
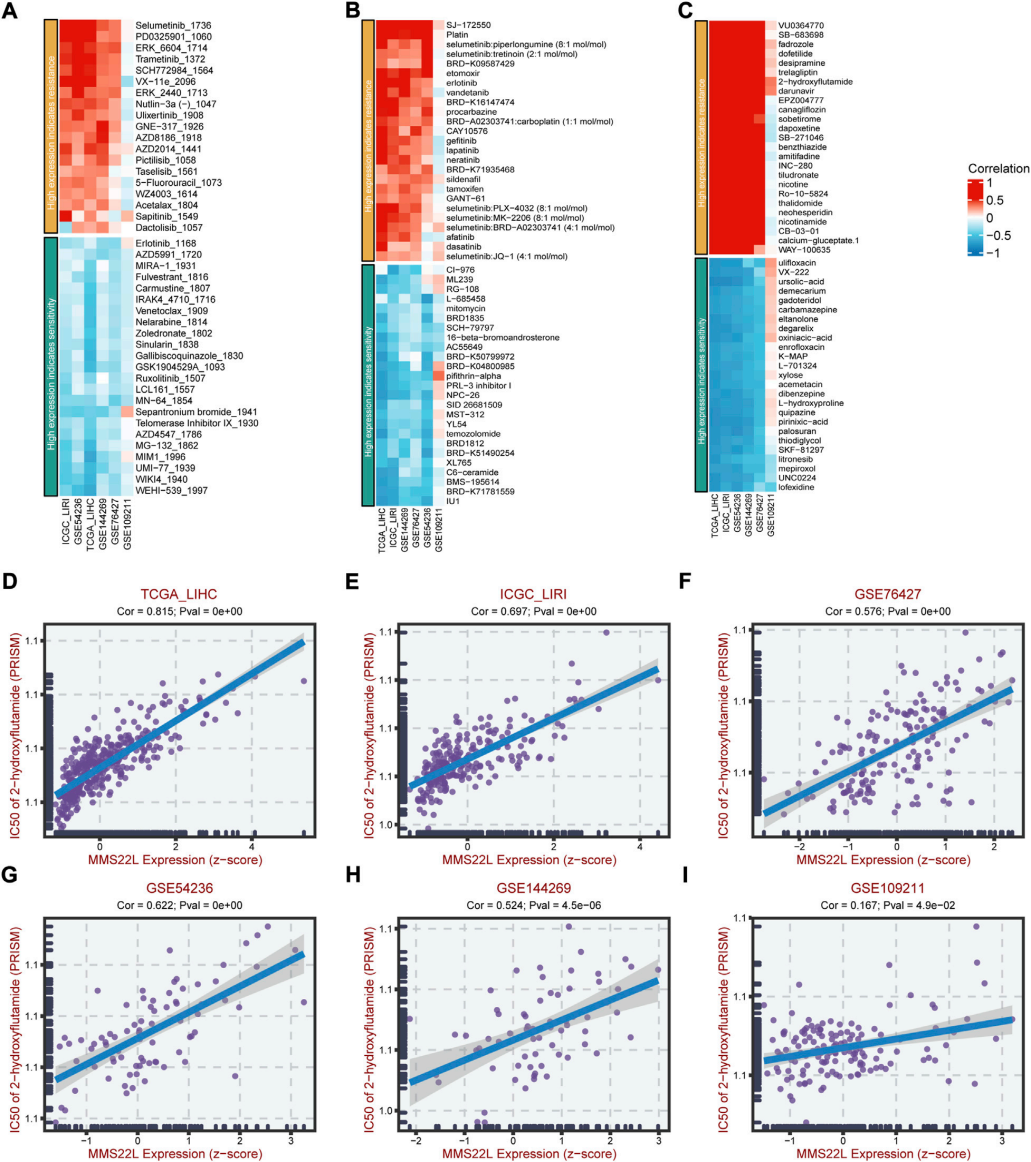
The association of methyl methanesulfonate-sensitivity protein 22-like (MMS22L) expression with drug sensitivity in hepatocellular carcinoma (HCC). **(A)** Relation of MMS22L expression with drug sensitivity in multiple HCC cohorts according to the GDSC2 database. **(B)** Relation of MMS22L expression with drug sensitivity in multiple HCC cohorts according to the CTRP database. **(C)** Relation of MMS22L expression with drug sensitivity in multiple HCC cohorts according to the PRISM database. **(D–I)** The correlation of MMS22L expression and 2-hydroxyflutamide sensitivity (IC50) in TCGA-LIHC **(D)**, ICGC-LIRI **(E)**, GSE76427 **(F)**, GSE54236 **(G)**, GSE144269 **(H)** and GSE109211 **(I)** according to the PRISM database.

Since drug resistance has been reported to have a strong relationship with various cancers and affects the prognosis and survival of cancer patients, we further studied the clinical manifestations of MMS22L in HCC. We further studied the relationship between MMS22L expression and drug resistance in HCC. We analyzed the correlation between the MMS22L high expression group and the drug resistance and sensitivity to different drugs in three databases, GDSC2, CTRP, and PRISM, in six HCC cohorts. The results showed that in these three databases, particularly PRISM, the high expression of MMS22L positively correlated with the drug resistance to most chemotherapeutic drugs. On the contrary, the high expression of MMS22L was highly negatively correlated with drug sensitivity, which indicated that the high expression of MMS22L might be the reason for the low sensitivity of HCC cells to certain chemotherapeutic drugs, thus affecting the prognosis and survival of patients (Figures 9A–C). Next, we tested the drug 2-hydroxyflutamide, which showed a high correlation with MMS22L expression in the six cohorts, and the results indicated that the IC50 value of this drug was highly correlated with MMS22L expression in all six cohorts (Figures 9D–I). This showed that MMS22L might be a potential target to improve the suppression of HCC.

## Discussion

MMS22L is widely expressed in human tumor tissues and plays different roles in the occurrence and development of various cancers, thus highlighting the possibility of MMS22L as a potential target for cancer therapy. Previous reports mainly identified MMS22L as a potential oncogene, which enhanced its nuclear localization and stability by binding to NFKBIL2 to promote tumor cell growth (Nguyen et al., 2012). Another study reported that MMS22L promoted tumor progression as a biomarker of breast cancer bone metastasis (Savci-Heijink et al., 2016). Nevertheless, not all reports suggested that MMS22L functioned as a cancer-promoting factor, promoting the occurrence and development of cancer. Recent studies have shown that low expression of MMS22L is associated with poor survival and lymph node metastasis and enhances tumor cell migration in ESCC (Luo et al., 2021), suggesting the feasibility of MMS22L as a tumor suppressor gene. However, the function of MMS22L in the majority of human tumors is yet to be thoroughly investigated. Bioinformatics analysis has made the understanding of high-throughput data completer and more strengthened the current understanding of tumor diseases (He et al., 2021; Qiu et al., 2021; Shen et al., 2022). In the current study, we characterized the landscape of MMS22L in pan-cancer through a comprehensive analysis of public repository data. We characterized the hallmark of MMS22L and preliminarily explored the potential mechanism of action of MMS22L and its association with the prognosis of tumor patients, immune cell infiltration, and genomic heterogeneity in pan-cancer. Concomitantly, the evaluation effect of MMS22L in immunotherapy was also shown in this study. More importantly, we identified the feasibility of MMS22L as a molecular marker in HCC by analyzing multiple HCC cohorts. These results described the critical role of MMS22L in human cancers while highlighting the feasibility of MMS22L as a potential therapeutic target for HCC. Altogether, these findings provide useful insights into the value of MMS22L in human cancer therapy.

It is well known that DNA replication plays an irreplaceable role during the cell division process. In humans, genome duplication largely depends on HR (Lopes et al., 2010). Although previous studies have reported that MMS22L is crucial in mediating HR and affecting genome duplication (Saredi et al., 2016), its expression pattern in tumors and its genomic mutational signature remain uncharacterized. In the present study, we first analyzed the landscape characteristics of MMS22L in pan-cancer. Our findings showed that MMS22L had a broad expression pattern in most tumor types and was abnormally expressed in most tumor tissues, indicating the potential of MMS22L as a tumor marker. Next, since the prognosis of tumor patients remains the most concerning issue in cancer research, some studies have characterized the critical role of MMS22L in tumors and its relationship with prognosis. In different omics data, the description of the survival correlation between MMS22L and patients with cancer is still lacking. Therefore, we further explored the association of MMS22L with clinical outcomes. Overall, the effects of MMS22L on the prognosis of patients with different tumor types at different molecular levels were significantly different. At the transcriptional level, the mRNA expression levels of MMS22L appeared to play completely different roles in different tumors. In previous studies, a higher expression of MMS22L in tumor tissues of ESCC was associated with better survival. However, no correlation was shown in our study, and this difference may be due to the insufficient sample size and esophageal cancer histological type in other studies. At the genomic DNA level, MMS22L with deletion or amplification in CNV was associated with worse OS. Previous studies have speculated that MMS22L mutations caused genomic instability and promoted certain types of ovarian and breast cancer (Piwko et al., 2016); however, our data suggested that patients with MMS22L mutations had a better prognosis than patients with wild-type MMS22L in UCEC. We believe that this does not contradict our observations. In normal cells, aberrant alterations of MMS22L lead to genomic instability and malignant transformation. However, in tumor cells, MMS22L mutations persist throughout tumor development. Since tumor cells divide significantly more frequently than normal cells and have a great demand for DNA damage repair, MMS22L mutations may limit the ability of the tumor to replicate, thereby inhibiting tumor progression. In addition, lack or inhibition of DNA damage repair can increase the sensitivity of tumor cells to DNA-damaging drugs and thus help overcome chemoresistance. Nonetheless, these results suggested that MMS22L produced in molecular modification events at different genetic levels had different prognostic effects on patients with different types of tumors. An in-depth analysis of different omics can help us better judge the prognosis of tumor patients.

The previous analytical results suggested that MMS22L played an essential role in tumor progression and prognosis; however, its mechanism of action remains unclear. Abnormal expression of proto-oncogenes or tumor suppressor genes at the transcriptional level has been widely reported as a key factor in tumor activation in previous studies. Therefore, we grouped tumor samples according to MMS22L mRNA expression levels and performed GSEA analysis to explore the role and potential mechanism of MMS22L in pan-cancer. GSEA results suggested that MMS22L was closely associated with activating cell cycle-related processes in tumors, including mitosis, cellular G2M checkpoints, and targeting the transcription factor E2F. These results suggested an important effect of MMS22L on cell proliferation; however, these results were expected since previous results had shown MMS22L as a key factor in the occurrence of HR events in cells. As an essential factor of cell proliferation and division, it is understandable that MMS22L participates in cell cycle regulation. Overall, these results also further affirm the reliability of the analytical results of this study. However, as mentioned in the results, we observed significant but conflicting effects of MMS22L on specific tumor immune response pathways in different cancers. These results also suggested that MMS22L plays a role in immune regulation in some specific tumor types. However, further research is needed to determine whether MMS22L performs these functions.

MMS22L has been widely reported to play an important role in the HR repair. However, since aberrant alterations in HR-related genes lead to an increased susceptibility to cancer, HR repair deficiency has been observed in nearly all cancer types (van Wilpe et al., 2021). Furthermore, although HRD promotes short-term benefits by increasing tumor sensitivity to chemotherapy, all patients eventually develop resistance to these therapies. Therefore, it is necessary to identify treatment options with more durable efficacy. HRD tumors are thought to be more immunogenic and, therefore, more amenable to treatment with checkpoint inhibitors. Concurrently, a previous study also showed that MMS22L might encode a cancer-testis antigen since the cancer-testis antigen is strongly expressed in tumors and has strong immunogenicity and antigen specificity. Therefore, its importance in tumor immunotherapy has gradually gained attention. These shreds of evidence point to the possibility of MMS22L assisting immunotherapy. Previous studies and reports on MMS22L and immune cells in the tumor microenvironment are still rare. Therefore, in this study, we first explored the correlation between the expression level of MMS22L and immune cell infiltration in the human tumor microenvironment and further compared the role of MMS22L in different immunotherapy cohorts through existing clinical studies. Similarly, and consistent with previous functional analyses, MMS22L exhibited conflicting responses in different immunotherapy cohorts. These disparate results might be due to the different genomic compositions of tumors or the different immune microenvironments between different tumor types. In addition, since cancer-testis antigens can induce specific cellular and humoral immune responses, there are currently two different strategies for using cancer-testis antigens as tumor immunotherapy targets: one is to directly enhance cancer-testis antigen-specific T lymphocytes, known as adoptive T-cell therapies (Xia et al., 2018; Zhang and Wang, 2019), and the other is to introduce other cancer-testis antigens to promote immune recognition and enhance anti-tumor immune responses (Dreno et al., 2018; Palata et al., 2020). Although this study showed some associations between MMS22L and immune cell infiltration, it still seemed insufficient to explain the direct impact of MMS22L on immunotherapy. We believe that introducing other cancer-testis antigens is likely to be an important way for MMS22L to participate in immunotherapy. Concomitantly, previous studies have described MMS22L in detail as an important member of the protein interaction network. Therefore, we believe that the role of tumor vaccine after introducing other cancer-testis antigens is likely to be the fundamental role of MMS22L in tumor immunotherapy. Overall, on the one hand, these results suggest that previous studies using a single immunotherapy cohort to evaluate potential markers may have certain limitations in understanding the role of tumor markers in immunotherapy. On the other hand, these results suggest a possible link between MMS22L and tumor immunotherapy. Targeting MMS22L may contribute to the development of tumor vaccines.

HCC, the most common type of liver cancer, has an increasing incidence worldwide (Carcinoma, 2021). The application of targeted drugs, such as sorafenib and combination therapeutic approaches, has brought hope for the treatment of patients with HCC (Llovet et al., 2008; Bruix et al., 2017; El-Khoueiry et al., 2017) however, it remains significant to identify effective prognostic markers and further improve clinical outcomes. MMS22L mRNA expression levels are significantly associated with patients’ prognoses in previous results. However, the clinicopathological significance of MMS22L in HCC remains unknown, and no studies have elucidated the role of abnormal expression of MMS22L mRNA in developing HCC. Therefore, our study further selected the role of MMS22L in HCC for further analysis. It can be seen that, as the degree of malignancy increased, MMS22L expression increased in HCC. Concurrently, the analytical results of multiple independent cohorts showed that MMS22L was highly expressed in HCC tissues, and these high expressions were significantly associated with patients’ poor prognoses. Further univariate and multivariate Cox regression analytical results also proved that MMS22L was an independent factor affecting the prognosis of patients with HCC. These results further confirm the reliability of the findings of this study. To further determine the practical application value of MMS22L in clinical treatment, this study analyzed the relationship between MMS22L expression and drug sensitivity. The role of MMS22L as a drug sensitivity regulator has been reported in various tumors. Reducing drug resistance and increasing drug sensitivity are of great significance as key issues in current clinical treatment. In this study, we focused on the significant association between the drug sensitivity of 2-hydroxyflutamide and MMS22L in HCC. The effect of 2-hydroxyflutamide as an antiandrogen has been reported in previous studies. Meanwhile, the use of antiandrogens to treat HCC patients has been extensively reported (Zhang et al., 2021). However, the related results are controversial. *In vitro* studies have shown that the antiandrogens cyproterone acetate and flutamide have inhibitory effects on androgen-induced HCC cell growth (Koch et al., 2015; Chen et al., 2021). In contrast, large clinical trials with leuprolide and flutamide (antiandrogens) have failed to improve patient survival (GdEedTdC, 2004). These results suggest that the effects of androgens or antiandrogens on the progression of HCC are yet to be further explored. The clinical application of targeted androgens in the treatment of HCC is feasible; however, there are still some limitations. In this study, a high expression of MMS22L resulted in resistance to 2-hydroxyflutamide, suggesting that targeting MMS22L may be a strategy to improve the current androgen-targeted suppression of HCC. These results provide a reference research direction for MMS22L; however, the actual role of MMS22L still needs to be explored by further experiments.

Although this study characterizes the role of MMS22L in pan-cancer and reveals an important role of MMS22L in HCC, there are inevitably some limitations in this study. The pan-cancer data in this study is dominated by the TCGA dataset, which is systematically biased. Furthermore, although we used as many cohorts as possible to elucidate the important role of MMS22L in HCC, expanding the cohort or in-depth mechanistic experiments may help us better understand the role of MMS22L in HCC. To our knowledge, this is the first study to provide a comprehensive description of MMS22L and preliminarily demonstrate its association with important research data in various tumors. It also demonstrates the potential mechanism of MMS22L in pan-cancer and its potential function in predicting immunotherapy response. Furthermore, MMS22L plays a key role in tumor progression and is associated with poor prognosis in patients with HCC, highlighting the therapeutic and diagnostic potential of MMS22L in HCC. We hope this pan-cancer analysis of MMS22L will help guide basic, translational, and clinical research targeting MMS22L in human cancers.

## Data Availability

The original contributions presented in the study are included in the article/Supplementary Material further inquiries can be directed to the corresponding author.

## Abbreviations

TMB, tumor mutational burden; MSI, microsatellite instability; HRD, homologous recombination deficiency; LOH, loss of heterozygosity; TCGA, The Cancer Genome Atlas; GEO, gene expression omnibus; ICGC, International Cancer Genome Consortium; CNV, copy number variation; GSEA, gene set enrichment analysis; NR, nonresponse; R, response; HR, homologous recombination; OS, overall survival; DSS, disease-specific survival; DFI, disease-free interval; PFI, progression-free interval.
