# Supplementary figures and images for: Integrative pan-cancer landscape of MMS22L and its potential role in hepatocellular carcinoma
Source: Front Genet. 2022 Oct 6;13:1025970. doi: 10.3389/fgene.2022.1025970 (PMC9582350; doi:10.3389/fgene.2022.1025970)

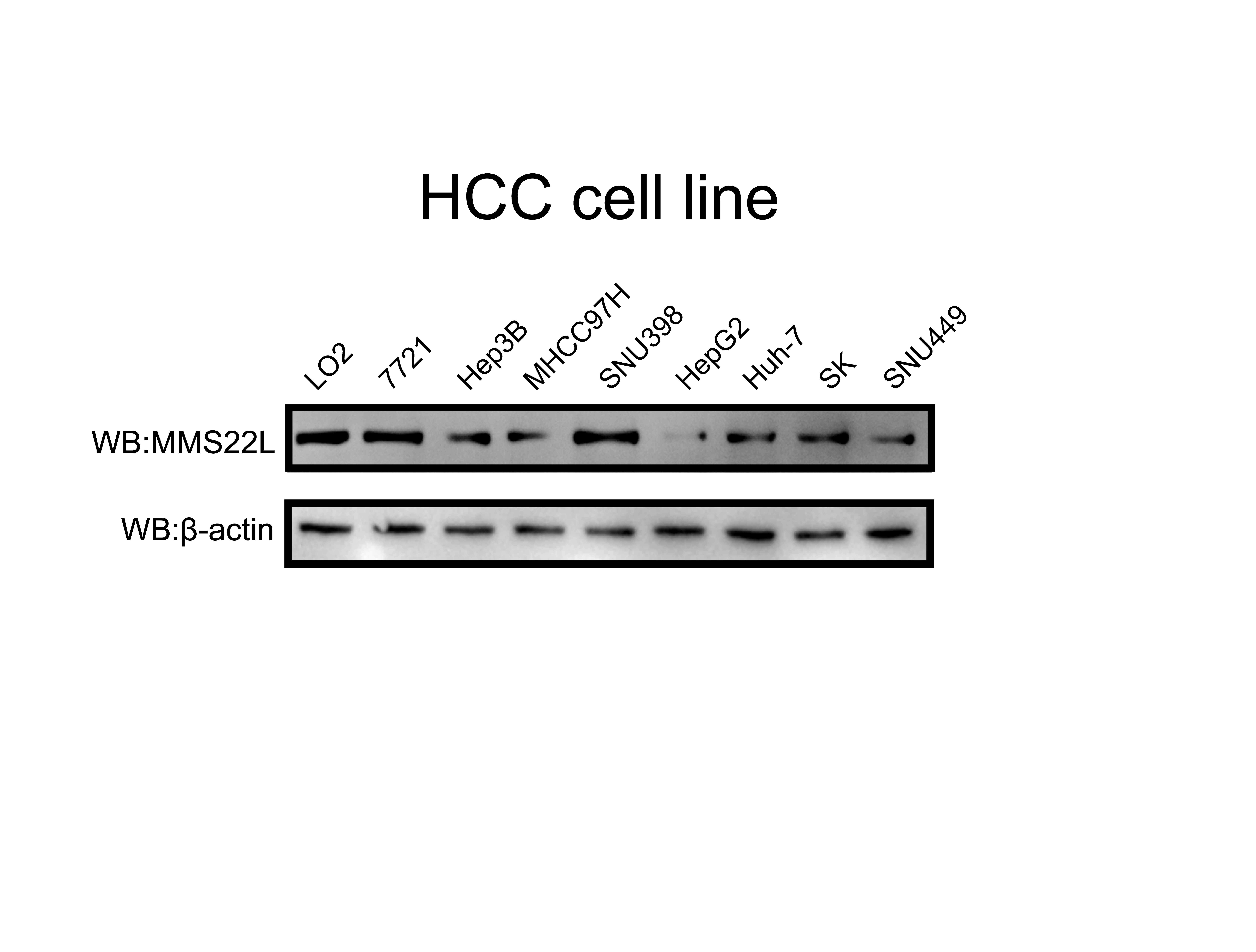

Supplement: Supplementary file 1 [file Image2.TIF]

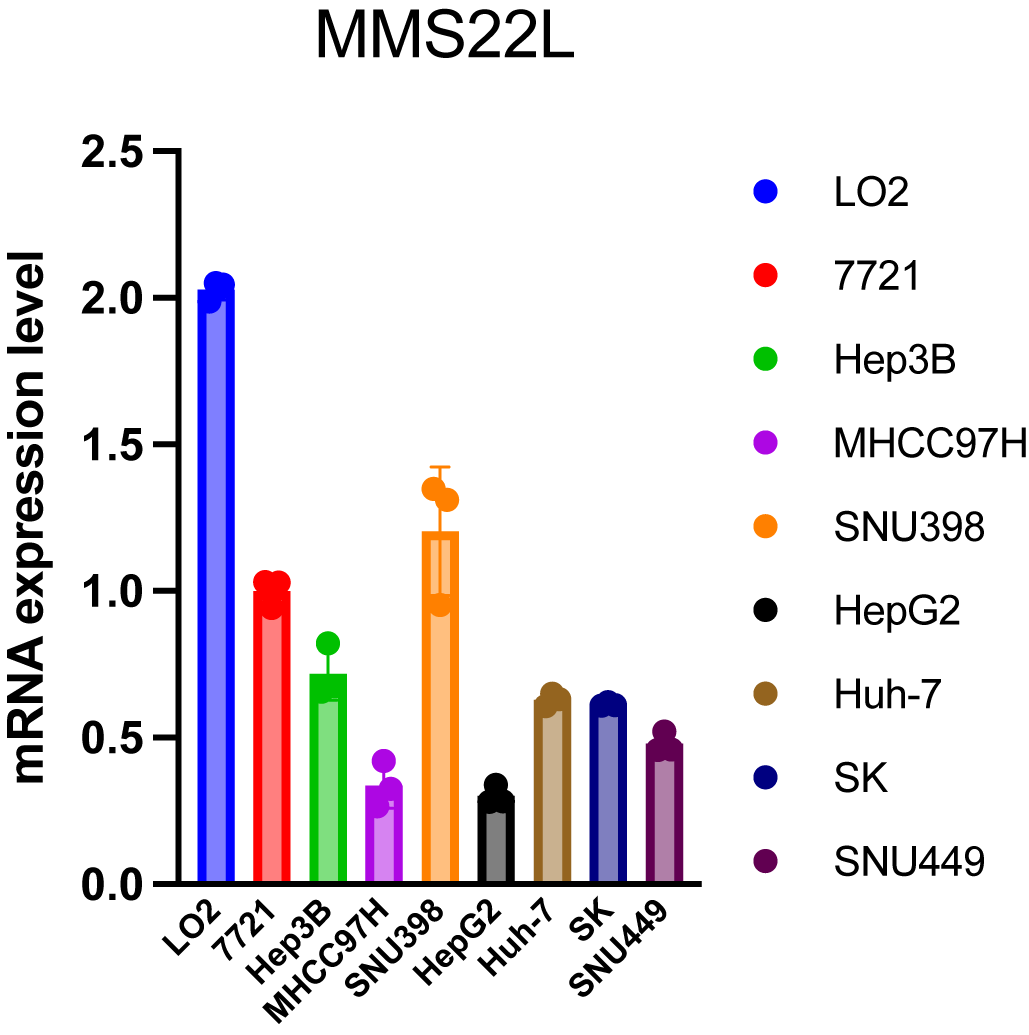

Supplement: Supplementary file 2 [file Image1.TIF]
